# Effectiveness of Local Use of Green Propolis-Loaded Lipid Nanoparticles as Adjuvant Therapy to Scaling and Root Planing in the Management of Periodontitis in Rats Treated with Zoledronate

**DOI:** 10.3390/ijms252212443

**Published:** 2024-11-20

**Authors:** Glauco Rodrigues Carmo Silveira, Vinícius Franzão Ganzaroli, Luan Felipe Toro, Estevão Lopes-Pereira, Leandro Lemes da Costa, João Martins de Mello-Neto, Rogério Leone Buchaim, Valdir Gouveia Garcia, Leticia Helena Theodoro, José Maurício Sforcin, Priscyla Daniely Marcato, Edilson Ervolino

**Affiliations:** 1 Department of Basic Sciences, School of Dentistry, São Paulo State University (UNESP), Araçatuba 16015-050, SP, Brazil; glauco.silveira@unesp.br (G.R.C.S.); vinicius.ganzaroli@unesp.br (V.F.G.); luan.toro@unesp.br (L.F.T.); estevao.pereira@unesp.br (E.L.-P.); leandro.lemes@unesp.br (L.L.d.C.); 2 Department of Diagnostic and Surgery, School of Dentistry, São Paulo State University (UNESP), Araçatuba 16015-050, SP, Brazil; leticia.theodoro@unesp.br; 3 Institute of Biosciences, São Paulo State University (UNESP), Botucatu 18618-000, SP, Brazil; jose.m.sforcin@unesp.br; 4 Marília Medical School (FAMEMA), Marília 17519-030, SP, Brazil; 5 College of Medicine and Dentistry, James Cook University, Cairns, QLD 4870, Australia; joao.martinsdemelloneto@jcu.edu.au; 6 Department of Biological Sciences, Bauru School of Dentistry, University of São Paulo (USP), Bauru 17012-901, SP, Brazil; rogerio@fob.usp.br; 7 Latin American Institute of Dental Research and Education (ILAPEO), Curitiba 80810-030, PR, Brazil; valdir.garcia@unesp.br; 8 Department of Pharmaceutical Sciences, School of Pharmaceutical Sciences, University of São Paulo (USP), Ribeirão Preto 14040-903, SP, Brazil; pmarcato@fcfrp.usp.br

**Keywords:** nanoparticles, periodontitis, propolis, scaling and root planing, zoledronate

## Abstract

This study assessed the effectiveness of the local use of green propolis-loaded lipid nanoparticles (GPlnp) as an adjuvant therapy to scaling and root planing (SRP) to manage experimental periodontitis (EP) in ovariectomized rats treated with zoledronate. Ten weeks before the experiment, 48 female rats were ovariectomized. On day 0, a ligature was installed in the lower first molar to induce EP. From day 0 to day 42, half of the rats were treated with vehicle (VEH), while the other half were treated with 100μg/Kg of zoledronate (ZOL). On day 14, the rats were allocated into the following groups: VEH-NLT, VEH-SRP, VEH-SRP-GPlnp, ZOL-NLT, ZOL-SRP, and ZOL-SRP-GPlnp. VEH-NLT and ZOL-NLT received no local treatment. VEH-SRP and ZOL-SRP received SRP and irrigation with physiological saline solution. VEH-SRP-GPlnp and ZOL-SRP-GPlnp received SRP and irrigation with GPlnp. A single SRP session was carried out, and four irrigation sessions were conducted (on days 14, 16, 18, and 20). On day 42, all animals were euthanized. The hemimandibles were processed for histological, histometric (percentage of total bone tissue (PTBT) and non-vital bone tissue (PNVBT)) and immunohistochemical (TNFα, IL-1β, and TRAP) analysis. VEH-SRP-GPlnp showed better tissue repair, higher PTBT, and lower immunolabeling for TNFα and IL-1β compared to the groups treated with VEH. ZOL-SRP-GPlnp showed a favorable tissue repair, with lower PNVBT, less local inflammation, and lower immunolabeling for TNFα and IL-1β compared to the groups treated with ZOL. Irrigation with GPlnp proved to be effective as an adjuvant therapy to SRP in treating EP in ovariectomized rats treated with zoledronate.

## 1. Introduction

Medication-related osteonecrosis of the jaws (MRONJ) is characterized by the presence of nonhealing exposed bone, or bone that can be probed through an intraoral or extraoral fistula in the maxillofacial region, that has persisted for more than eight weeks, in patients with previous or current treatment with antiresorptive and/or antiangiogenic drugs and no history of radiotherapy or metastatic disease in the jaws [1]. The etiopathogenesis of MRONJ is not fully understood. It has been shown that bisphosphonates potently suppress osteoclasts’ resorptive activity, leading to an accumulation of microdamage to bone tissue. These drugs also exhibit an antiangiogenic effect, cytotoxic action, causes dysfunction in the local immune response and increase the susceptibility to infection [2].

The presence of MRONJ significantly compromises life quality and can lead to debilitating consequences if the disease progresses [3]. MRONJ treatment can be lengthy and often unsuccessful and is determined based on the disease’s staging. It may involve a combination of surgeries, ranging from curettage to partial jaw resection, and long-term use of antimicrobial drugs [4,5].

The main systemic risk factor for MRONJ is the use of antiresorptive drugs at oncological dosage. In addition, invasive dental procedures such as tooth extractions account for most cases [6,7,8]. Clinical studies have indicated that periodontitis is the second most significant risk factor associated with the disease [9,10,11]. Other authors used animal models to confirm the associations between periodontitis and MRONJ [12,13,14]. Thus, it is crucial to manage periodontitis while undergoing treatment with antiresorptive drugs effectively. The standard treatment for periodontitis is scaling and root planing (SRP), which promotes the removal of bacterial deposits and plaque-retentive factors, effectively reversing and stabilizing periodontitis [15,16]. Similarly, SRP is effective in treating experimental periodontitis (EP) in an experimental rat model [17,18,19].

The occurrence of MRONJ after treatment with SRP has been reported in patients receiving antiresorptive drugs [20,21]. Araújo et al. [22] reported that EP treatment with SRP did not alter alveolar bone loss in rats. However, it caused a prolonged exacerbation of the local inflammatory response and considerably increased the amount of non-vital bone tissue consistent with stage 0 of MRONJ. These findings underscore the importance of effectively managing periodontal disease during or shortly after treatment with antiresorptive drugs with minimally invasive therapies. While these treatments may not always be effective, they can be combined with other therapies. It is beneficial to use treatments that regulate local inflammation, stimulate periodontal tissue, and provide antimicrobial action to ensure effectiveness and safety.

It has been shown that different types of propolis associated with SRP may be natural and effective treatment options for periodontitis [23,24,25]. Propolis is a resinous material that bees collect from plant buds and exudates and mix with bee enzymes, pollen, and wax. Propolis’s composition varies depending on the profile of active components due to the botanical and geographical natures of the region in which it was produced. In general, its composition contains resin and balms (50–60%), wax, and essential aromatic oils (30–40%) mixed with bee salivary secretion (10%), pollen (5%), and other substances (5%), such as amino acids, minerals, and vitamins [26]. In Brazil, particularly in the southeast region, green propolis is primarily produced from *Baccharis dracunculifolia*, which explains its abundance in cinnamic acid derivative compounds, such as caffeic acid, *p*-coumaric acids, drupanin, artepelin C, and baccarin, in addition to flavonoids and phenolic acid [27]. Green propolis displays several biological effects such as anti-inflammatory, antioxidant, antimicrobial, immunomodulatory and biostimulatory actions in tissue repair, what justifies the investigation of its action in the management of periodontitis [28,29].

Nanotechnology-based delivery of substances is a growing research field. Nanostructured carrier systems can incorporate or improve the physicochemical properties of active ingredients, such as improving solubility, increasing bioavailability, providing protection against degradation, and enhancing diffusibility and permeability [30,31,32]. Recent preclinical studies have shown that nanocarrier systems of pharmaceuticals and other substances significantly advance the management of periodontitis [33]. Such systems can carry one or more substances that add to periodontitis treatment through antimicrobial activity and/or immunomodulatory action and/or stimulation of periodontal tissue repair [33]. The greater the ability of substances to achieve this triad of effects, the more promising the treatment results. In this context, green propolis, with its numerous properties, is an excellent option when delivered through a nanocarrier system. One of the options for delivering green propolis is through microemulsion-type nanocarrier systems. These systems consist of two immiscible liquids and one or more surfactants, which allow for one liquid to be dispersed in the other as nanometer-scale spheres [34,35].

The treatment of periodontitis during and after the use of antiresorptive drugs is challenging due to the lack of evidence regarding its effectiveness and potential MRONJ risks. Therefore, searching for effective and safe protocols is still necessary. The adjuvant use of propolis has already demonstrated effectiveness in treating periodontitis, but its application using a nanostructured delivery system has not been evaluated yet in this context, which could be capable of improving substantially its effectiveness. Therefore, this study aimed to assess the effectiveness and safety of using green propolis-loaded lipid nanoparticles (GPlnp) locally as an adjunct therapy to scaling and root planing (SRP) in managing experimental periodontitis (EP) disease in ovariectomized rats treated with zoledronate. To achieve this, a treatment protocol was developed using a high dose of zoledronate, a medication known for its strong antiresorptive properties. An experimental model of ligature-induced periodontitis was used in rats that had previously undergone ovariectomy. Once EP was established, the following two treatment modalities were established: standard, with SRP, and experimental, using GPlnp as an adjuvant therapy to SRP. After a period of post-treatment of EP, we conducted clinical, histological, histometric, and immunohistochemical analyses. The combination of SRP with the local use of GPlnp proved to be both effective and safe in the management of periodontitis in ovariectomized rats treated with an oncological dosage of zoledronate.

## 2. Results

### 2.1. Physicochemical Characterization of GPlnp

**Figure 1:**
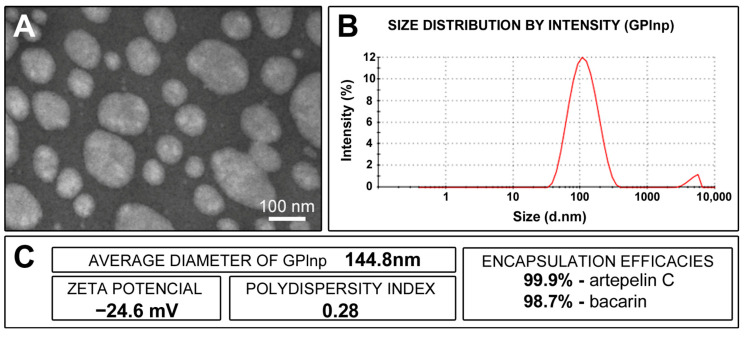
Characterization of lipid nanoparticles containing green propolis: (**A**) appearance of lipid nanoparticles under a transmission electron microscope; (**B**) graph showing the size distribution of lipid nanoparticles (in nanometers) after 90 days of preparation; (**C**) average diameter of the lipid nanoparticles, zeta potential, polydispersity index, and artepelin C and baccarin encapsulation efficacies.

The GPlnp had an average diameter of 144.8 nm, a zeta potential of −24.6 mV, a polydispersity index of 0.28, and encapsulation efficacies of 99.9% for artepelin C and 98.7% for baccarin (Figure 1).

### 2.2. Availability of General Health and Intraoral Condition of the Animals

The animals effectively managed all experimental procedures and maintained good health throughout the experimental period. This included ovariectomy, treatment with the vehicle and zoledronate, ligature placement, SRP, and adjuvant therapies. Throughout the experiment, the average body weight of the animals remained consistent, with no differences within or between the groups.

### 2.3. Histopathological Analyses of Periodontal Tissues

Less favorable histological characteristics were found in the VEH-NLT group, while the VEH-SRP-GPlnp group exhibited more favorable characteristics in the periodontal tissues. Regarding the groups treated with zoledronate, the ZOL-SRP-GPlnp group showed more favorable histological characteristics in the periodontal tissues. The ZOL-NLT and ZOL-SRP groups presented histological characteristics of Stage 0 MRONJ. Table 1 presents the parameters, scores, and distribution of the specimens based on the histopathological analyses of periodontal tissues in the lower-first molar region of the experimental groups.

### 2.4. PTBT

**Figure 2:**
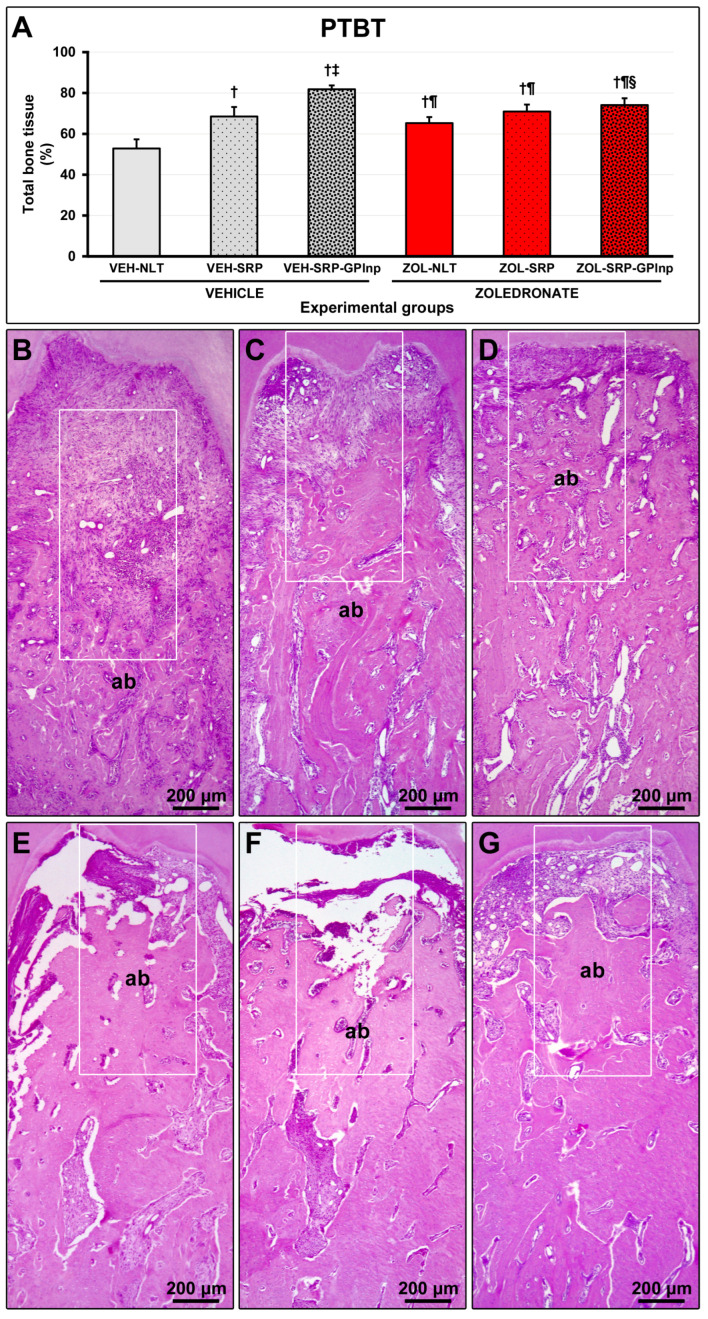
Bone tissue in the furcation area of the lower first molar: (**A**) graph showing the percentage of total bone tissue (PTBT) in the experimental groups; (**B**–**G**) photomicrographs showing the histopathological characteristics in the furcation region in the (**B**) VEH-NLT, (**C**) VEH-SRP, (**D**) VEH-SRP-GPlnp, (**E**) ZOL-NLT, (**F**) ZOL-SRP, and (**G**) ZOL-SRP-GPlnp groups. In the groups treated with vehicle, it was observed that local treatments were able to reduce alveolar bone loss, especially in the VEH-SRP-GPlnp group. Note that alveolar bone loss was not severe in the groups treated with zoledronate. In the ZOL-NLT and ZOL-SRP groups, there was significant disruption of the connective tissue and extensive impairment of the bone tissue vitality. This was not observed in ZOL-SRP-GPlnp, which presented a more favorable condition. The white rectangles delimit the regions that are presented in greater magnification in Figure 3. Statistical test: Shapiro–Wilk test and variance analysis (ANOVA) followed by the Tukey post-test. Abbreviations and symbols: ab, alveolar bone; †, a statistically significant difference compared to VEH-NLT; ‡, a statistically significant difference compared to VEH-SRP; ¶, a statistically significant difference compared to VEH-SRP-GPlnp; §, a statistically significant difference compared to ZOL-NLT. Staining: Hematoxylin and Eosin. Original magnification: 50×. Scale bars: 200 μm.

The VEH-NLT group (52.8 ± 4.5) showed lower PTBT than other experimental groups (VEH-SRP, 68.5 ± 4.7; VEI-SRP-GPlnp, 81.8 ± 1.9; ZOL-NLT, 65.2 ± 3.0; ZOL-SRP, 70.9 ± 3.4; and ZOL-SRP-GPlnp, 74.1 ± 3.3). The PTBT in the VEH-SRP-GPlnp group was higher than in the other experimental groups. The ZOL-SRP-GPlnp group showed higher PTBT compared to the ZOL-NLT group (Figure 2).

### 2.5. PNVBT

**Figure 3:**
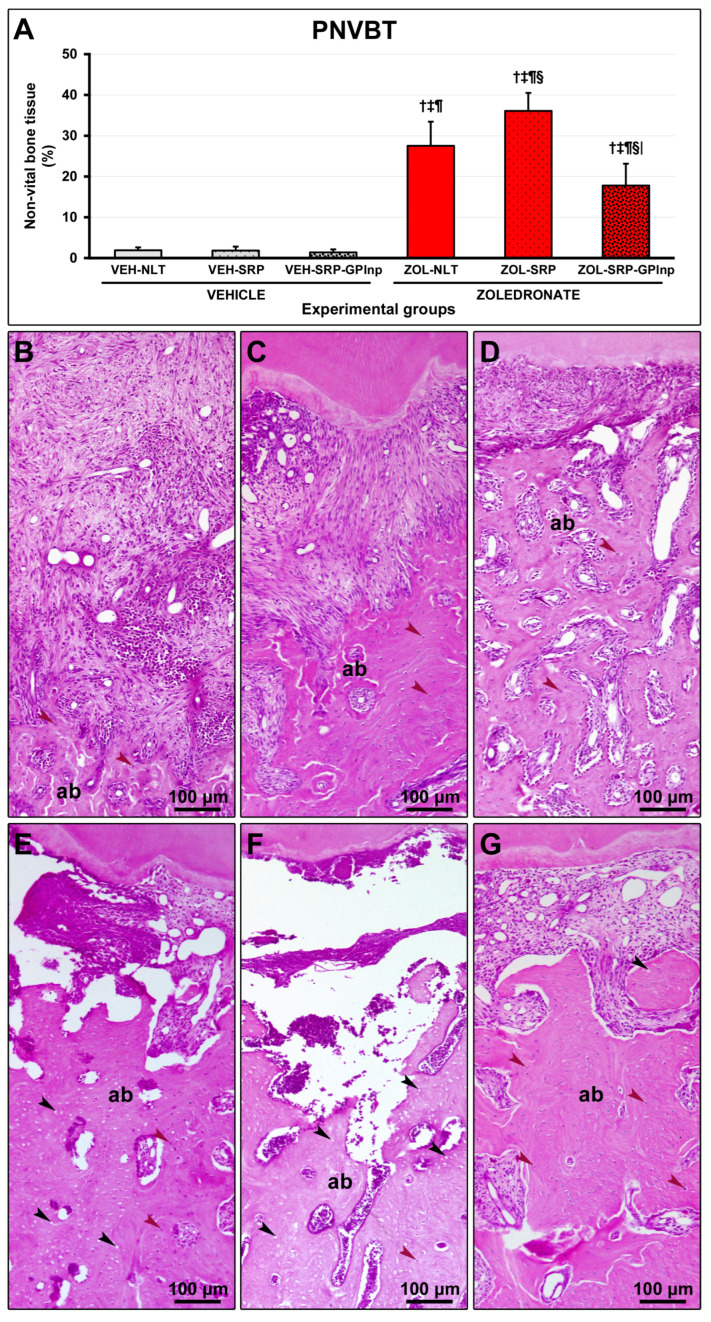
Non-vital bone tissue in the furcation area of the lower first molar: (**A**) graph showing the percentage of non-vital bone tissue (PNVBT) in the experimental groups; (**B**–**G**) photomicrographs showing the histopathological characteristics in the furcation region in the (**B**) VEH-NLT, (**C**) VEH-SRP, (**D**) VEH-SRP-GPlnp, (**E**) ZOL-NLT, (**F**) ZOL-SRP, and (**G**) ZOL-SRP-GPlnp groups. Note an intense inflammatory infiltrate in the VEH-NLT group. In contrast, the VEH-SRP and VEH-SRP-GPlnp groups presented better structured connective tissue, especially the latter. Note that in the ZOL-NLT and ZOL-SRP groups, the connective tissue was unstructured, and most of the bone tissue present was non-vital. In contrast, the ZOL-SRP-GPlnp group presented connective tissue with discrete inflammatory infiltrate, and the bone tissue presented few areas without vitality. Statistical test: Shapiro–Wilk test and variance analysis (ANOVA) followed by the Tukey post-test. Abbreviations and symbols: ab, alveolar bone; black arrows, empty lacunae; red arrows, osteocytes; †, statistically significant difference compared to VEH-NLT; ‡, statistically significant difference compared to VEH-SRP; ¶, statistically significant difference compared to VEH-SRP-GPlnp; §, statistically significant difference compared to ZOL-NLT; |, statistically significant difference compared to ZOL-SRP. Staining: Hematoxylin and Eosin. Original magnification: 100×. Scale bars: 100 μm.

PNVBT was higher in the zoledronate-treated groups (ZOL-NLT, 27.5 ± 5.9; ZOL-SRP, 36.1 ± 4.4; and ZOL-SRP-GPlnp, 17.8 ± 5.4) compared to the vehicle-treated groups (VEH-NLT, 1.9 ± 0.7; VEH-SRP, 1.8±1.0; and VEH-SRP-GPlnp, 1.4 ± 0.8). The ZOL-SRP group showed higher PNVBT compared to the ZOL-NLT group. PNVBT was lower in the ZOL-SRP-GPlnp group compared to the ZOL-NLT and ZOL-SRP groups (Figure 3).

### 2.6. Immunolabeling Density for TNFα and IL-1β

**Figure 4:**
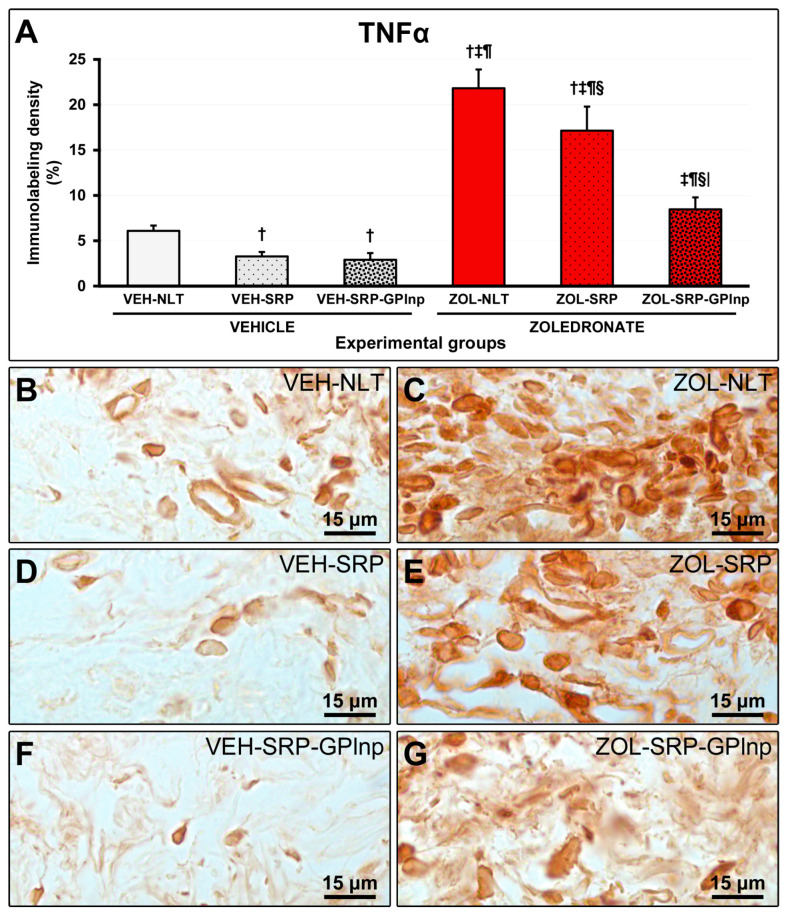
Immunolabeling for TNFα in the connective tissue of the furcation region: (**A**) Graphs showing the immunolabeling pattern for TNFα in the experimental groups. Statistical test: Shapiro–Wilk test and variance analysis (ANOVA) followed by the Tukey post-test. (**B**–**G**) Photomicrographs showing the immunolabeling patterns for TNFα in the mandibular first lower molar in the (**B**) VEH-NLT, (**C**) ZOL-NLT, (**D**) VEH-SRP, (**E**) ZOL-SRP, (**F**) VEH-SRP-GPlnp, and (**G**) ZOL-SRP-GPlnp groups. Note a higher density of immunolabeling for TNFα in the ZOL-NLT and ZOL-SRP groups. In the ZOL-SRP-GPlnp group, the local use of GPlnp as an adjuvant therapy to SRP resulted in a lower density of immunolabeling for TNFα. Symbols: †, statistically significant difference compared to VEH-NLT; ‡, statistically significant difference compared to VEH-SRP; ¶, a statistically significant difference compared to VEH-SRP-GPlnp; §, a statistically significant difference compared to ZOL-NLT; |, a statistically significant difference compared to ZOL-SRP. Original magnification: 1000×. Scale bars: 15 μm.

**Figure 5:**
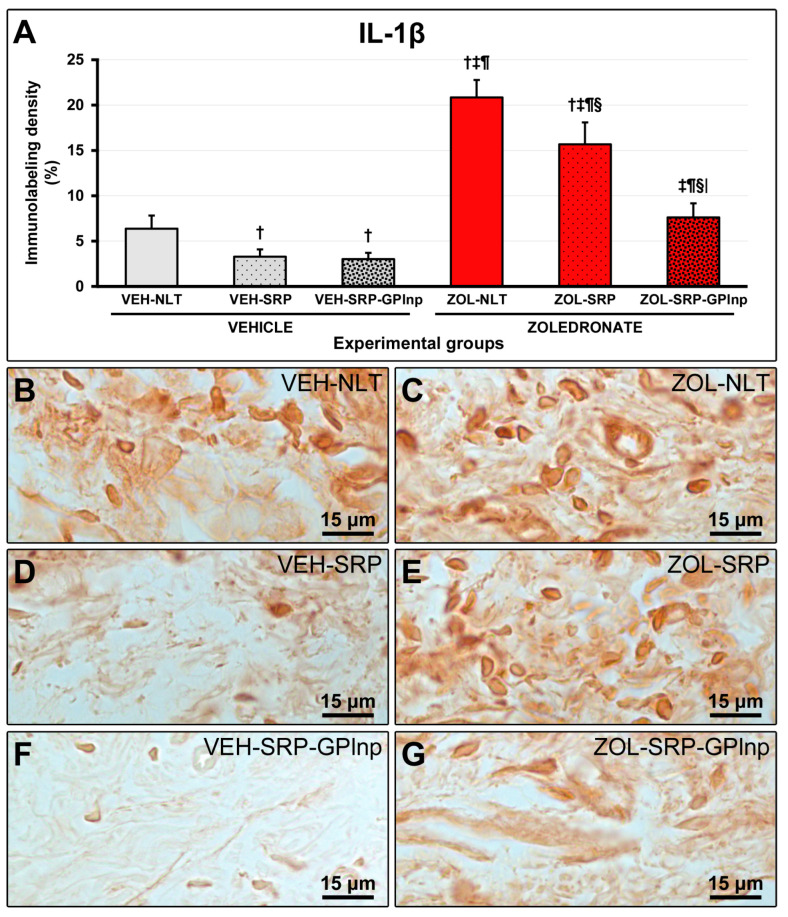
Immunolabeling for IL-1β in the connective tissue of the furcation region: (**A**) Graphs showing the immunolabeling pattern for IL-1β in the experimental groups. Statistical test: Shapiro–Wilk test and variance analysis (ANOVA) followed by the Tukey post-test. (**B**–**G**) Photomicrographs showing the immunolabeling patterns for IL-1β in the mandibular first lower molar in the (**B**) VEH-NLT, (**C**) ZOL-NLT, (**D**) VEH-SRP, (**E**) ZOL-SRP, (**F**) VEH-SRP-GPlnp, and (**G**) ZOL-SRP-GPlnp groups. Note the higher density of immunolabeling for IL-1β in the ZOL-NLT and ZOL-SRP groups. In the ZOL-SRP-GPlnp group, the local use of GPlnp as an adjuvant therapy to SRP resulted in a lower density of immunolabeling for IL-1β. Symbols: †, statistically significant difference compared to VEH-NLT; ‡, statistically significant difference compared to VEH-SRP; ¶, a statistically significant difference compared to VEH-SRP-GPlnp; §, a statistically significant difference compared to ZOL-NLT; |, a statistically significant difference compared to ZOL-SRP. Original magnification: 1000×. Scale bars: 15 μm.

Immunolabeling density for TNFα and IL-1β in the furcation region was lower in the vehicle-treated groups (TNFα: VEH-NLT, 6.1 ± 0.6; VEH-SRP, 3.3 ± 0.5; VEH-SRP-GPlnp, 2.9 ± 0.7; IL-1β: VEH-NLT, 6.4 ± 1.5; VEH-SRP, 3.3 ± 0.8; and VEH-SRP-GPlnp, 3.0 ± 0.7) compared to the zoledronate-treated groups (TNFα: ZOL-NLT, 21.8 ± 2.1; ZOL-SRP, 17.1 ± 2.7; ZOL-SRP-GPlnp, 8.5 ± 1.3; IL-1β: ZOL-NLT, 20.8 ± 1.9; ZOL-SRP, 15.7 ± 2.4; and ZOL-SRP-GPlnp, 7.6 ± 1.6), except for the VEH-NLT and ZOL-SRP-GPlnp groups, which showed no significant difference compared with each other. The VEH-NLT group showed a higher density of immunolabeling for TNFα and IL-1β compared to the VEH-SRP and VEH-SRP-GPlnp groups. The density of the immunolabeling for TNFα and IL-1β was lower in the ZOL-SRP-GPlnp group compared to the ZOL-NLT and ZOL-SRP groups (Figure 4 and Figure 5).

### 2.7. TRAP Immunolabeling

**Figure 6:**
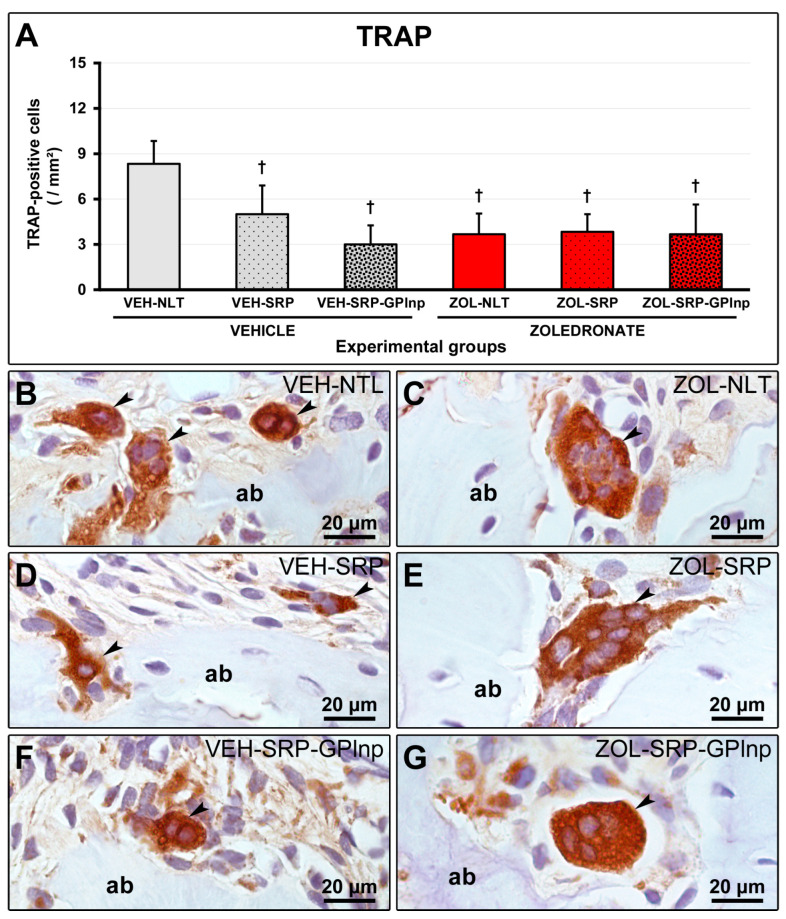
Immunolabeling for TRAP in the furcation region: (**A**) Graph showing the number of TRAP-positive cells in the furcation area of the experimental groups. Statistical test: Shapiro–Wilk test and variance analysis (ANOVA) followed by the Tukey post-test. (**B**–**G**) Photomicrographs showing the immunolabeling patterns for TRAP in the mandibular left molars in the (**B**) VEH-NLT, (**C**) VEH-SRP, (**D**) VEH-SRP-GPlnp, (**E**) ZOL-NLT, (**F**) ZOL-SRP, and (**G**) ZOL-SRP-GPlnp groups. Note the higher number of TRAP-positive cells in the VEH-NLT group. In the groups treated with zoledronate, observe that the TRAP-positive cells were larger than in the groups treated with vehicle, circumferential, hypernucleated, and distant from the bone matrix. Abbreviations and symbols: ab, alveolar bone; black arrows, TRAP-positive cells (osteoclasts); †, a statistically significant difference compared to VEH-NLT. Counterstain: Harris Hematoxylin. Original magnification: 1000×. Scale bars: 20 μm.

In the groups treated with zoledronate, TRAP-positive cells were larger than in the groups treated with vehicle, circumferential, hypernucleated, and distant from the bone matrix. The VEH-NLT group (8.3 ± 1.5) had a higher number of TRAP-positive cells in the furcation region of the lower first molar compared to the other experimental groups (VEH-SRP, 5.0 ± 1.9; VEI-SRP-GPlnp, 3.0 ± 1.3; ZOL-NLT, 3.7 ± 1.4; ZOL-SRP, 3.8 ± 1.2; and ZOL-SRP-GPlnp, 3.7 ± 2.0) (Figure 6).

## 3. Discussion

Antiresorptive drugs, although highly effective in treating bone conditions such as osteopenia/osteoporosis and osteolytic diseases, can lead to severe and debilitating side effects such as MRONJ [1]. In addition to the use of antiresorptive drugs, the development of MRONJ is also associated with the presence of local risk factors, which include invasive dental procedures such as dental extractions and an inflammatory–infectious process associated with the tooth, such as periodontitis [6,7,8]. SRP is considered the gold-standard treatment for periodontitis and is effective for most patients [15,16]. However, the response to SRP treatment may be lower depending on the severity of periodontitis and a compromised response of periodontal tissues to treatment. In patients undergoing treatment with zoledronate, especially at high dosages, these two situations may be compounded. Under these circumstances, adjuvant therapies may enhance treatment effectiveness and ensure necessary safety, mainly when a significant local risk factor for MRONJ is present. Therefore, the purpose of this study was to evaluate the effectiveness and safety of the local use of GPlnp as an adjuvant therapy to SRP in the treatment of EP in ovariectomized rats undergoing treatment with a high dose of zoledronate. Our results indicate that irrigation with GPlnp is effective and safe as an adjuvant therapy for SRP in this situation.

Experimental models have been developed to elucidate the periodontitis [36,37,38] and MRONJ [39,40] etiopathogeneses, being important for proposing and evaluating more targeted and efficacious treatment approaches. In this study, we used ovariectomized adult rats treated with an oncological dose of zoledronate since MRONJ affects post-menopausal women more frequently [6,7,8]. The use of zoledronate in the oncological dose was adopted because this drug, at this dosage, is associated with most cases of MRONJ [6,7,8]. Furthermore, the focus of attention of this study was periodontitis, which was induced by ligature, since it consists of the second local risk factor associated with MRONJ [9,10,11].

The primary outcome of this study was PNVBT. The use of this variable is based on the fact that bone necrosis is the hallmark of MRONJ [1,40]. Most studies proposing and evaluating treatment protocols for EP employ a primary outcome that reflects the amount of alveolar bone tissue lost or preserved. In the present study, we also used histometric analysis of PTBT. However, these constituted secondary outcomes since zoledronate promotes inhibition of the activity and number of the osteoclasts. As a result, the preservation of alveolar bone tissue may be greater in groups treated with zoledronate. However, these characteristics do not guarantee treatment success since the viability of this bone tissue may be severely compromised. Thus, it is essential to use a methodological tool, such as histopathological analysis, to ensure whether the bone tissue present is vital or non-vital.

In the groups treated with zoledronate, in addition to presenting a lower number of TRAP-positive cells than the VEH-NLT group, these osteoclasts presented histological characteristics very different from those shown in the groups treated with vehicle. The immunohistochemical analysis revealed that these cells were larger than usual, circumferential, hypernucleated, and distant from the bone matrix. These characteristics are typical of osteoclasts with compromised activity, indicating the influence of antiresorptive drug [41]. These findings justify the lack of statistical differences in PTBT between the ZOL-NTL and ZOL-SRP groups even in the face of exacerbated inflammation. In ZOL-SRP-GPlnp group, inflammation was modulated because of the treatment with GPlnp; consequently, there was even less bone resorption than in the other groups treated with zoledonate.

PNVBT in the ZOL-NLT and ZOL-SRP groups was significantly high at the EP site. Furthermore, a more severe and prolonged local inflammatory response was observed and confirmed with a high immunolabeling pattern for TNFα and IL-1β in the periodontal tissues. There is probably a close relationship between the intensity of inflammation and the death of osteocytes in the alveolar bone observed in the present study. Such findings of the present study could be explained based on the proposal of Aguirre et al. (2021) [40]. The progression of the inflammatory process in bone tissue may cause the death of osteocytes, predominantly through apoptosis or necroptosis. As the use of zoledronate efficiently inhibits bone resorption, the system that normally eliminates the remnants of old bone cells is unable to function correctly because it depends on the access achieved by the resorptive activity of osteoclasts. The death of osteocytes within the bone matrix generates damage-associated molecular patterns (DAMPs), of which SAP-130 stands out in necrotic alveolar bone. DAMPs access the surface of the bone matrix through lacunae and canaliculi and activate pattern recognition receptors (PRRs) expressed in osteoclasts. This includes osteoclasts affected by the use of zoledronate and cells with phagocytic capacity. Among the PRRs, Mincle is particularly prominent in alveolar bone tissue. The interaction between DAMPs and PRRs significantly amplifies the local inflammatory response, leading to increased osteocyte death, bone necrosis, and the creation of a positive inflammatory feedback loop [40].

None of the experimental groups showed any clinical signs of MRONJ. Supposedly, the clinical manifestation of MRONJ is associated with the amount of non-vital bone tissue present, and only when a certain threshold is exceeded does the clinical manifestation occur. One may speculate that if the EP induction period had been extended, this threshold could have been reached, as observed in a previous study [13]. The primary objective of this study was to propose adjuvant therapies aimed at preventing MRONJ as opposed to its treatment. Thus, our study design was appropriate and may be used in further studies assessing preventive strategies for MRONJ. Invasive dental procedures capable of generating inflammation in this site could also increase the risk of clinical manifestation of MRONJ. Some studies have demonstrated using a senescent rat model treated with an oncological dosage of zoledronate and submitted to extractions of first lower molars with EP presented with MRONJ, probably due to an exacerbation of the inflammatory response caused by the surgical procedure [42,43].

As mentioned earlier inflammation can lead to osteocyte death which can lead to secondary inflammation. Therefore, breaking this cycle is likely to have positive local effects. In the ZOL-SRP-GPlnp group, non-vital bone tissue was lower compared to the other groups treated with zoledronate. In addition, a significantly reduced inflammatory condition was observed, associated with decreased immunolabeling for TNFα and IL-1β. It has been demonstrated that green propolis possesses a potent anti-inflammatory effect, primarily due to its high concentration of artepelin C, baccarin and *p*-coumaric acid [44,45,46]. Probably, the anti-inflammatory action of the green propolis was responsible for breaking the inflammation cycle, reducing osteocyte death, and reducing the amount of non-vital bone tissue in this experimental group.

Although the antimicrobial activity of GPlnp was not the focus of this study, its capacity to reduce bacterial load [47,48,49], including periodontopathogenic microorganisms [50,51], may also be associated with reducing local inflammation. The antimicrobial effects of propolis can be attributed to several of its constituents, which can cause structural and/or functional damage to bacteria [52,53,54]. Some studies have reported that the effect of propolis is greater in Gram-positive bacteria [53,55]. Gram-negative bacteria have a chemically more complex cell wall with a higher lipid content than Gram-positive bacteria [56]. This difference could be able to offer greater protection for Gram-negative bacteria against the effects of propolis. In the present study, green propolis was used in a system composed of lipid nanoparticles. Supposedly the lipid nanoparticles easily overcome this lipid-based defense mechanism of this type of bacteria, and green propolis would exert its antimicrobial role at a maximum potency also in Gram-negative bacteria. However, potent effects of propolis and some of its constituents have been reported, including baccarin and artepelin C, on *Porphyromonas gingivalis*, a Gram-negative anaerobic bacterium that plays a key role in periodontitis [57]. The action of this bacteria is an important effect considering that zoledronate increases the susceptibility of periodontal tissues to infection by *Porphyromonas gingivalis* [58]. Therefore, at least part of the reduction in local inflammation observed in the ZOL-SRP-GPlnp group was probably also related to GPlnp antimicrobial effects.

Green propolis also possesses significant antioxidant properties, primarily attributed to the high concentration of flavonoids and phenolic compounds. Studies show an excessive production of reactive oxygen species throughout the progression of periodontitis [59] and also osteonecrotic lesions [60] and the inability of antioxidant systems to return to redox balance, leading to oxidative damage locally. Green propolis may play a valuable role in mitigating this oxidative stress due to its potent antioxidant capacity. Therefore, GPlnp could reduce local oxidative stress and subsequent damage, aiding the site in restoring itself or at least get closer to homeostasis.

GPlnp positively contributed to the results observed in the ZOL-SRP-GPlnp group compared to ZOL-SRP due to its biostimulatory action on the periodontal tissues repair process [61,62,63]. After SRP, periodontal tissues go through the following usual phases of tissue repair: the inflammatory phase, the proliferative phase, and the remodeling phase [64]. In the ZOL-SRP group, the intensity and persistence of the inflammatory phase may have compromised the entire tissue repair process. The biostimulatory action on some cell lineages attributed to the adjunctive use of green propolis during the modulation of inflammation and in the proliferative phase of the tissue repair process would be capable of bringing periodontal health, as observed in the ZOL-SRP-GPlnp group.

MRONJ is a condition whose treatment represents a significant challenge in dentistry. Strategies preventing such a condition would be ideal instead of its treatment. In addition to the systemic risk factors, such as the use of antiresorptive drugs, a local risk factor, such as periodontitis, is generally necessary for MRONJ occurrence. Thus, before starting treatment with antiresorptive drugs, a complete oral health assessment is advisable. If periodontitis develops during treatment with this type of drug, it must be treated, as it constitutes a risk factor for MRONJ. Conventional treatment with SRP should minimize tissue damage to avoid iatrogenic factors. In addition, adjuvant therapies may be suggested to achieve a satisfactory periodontal condition and reduce tissue damage. The use of GPlnp constituted an adjuvant therapy that mitigated the local inflammatory condition and, consequently, limited the negative consequences of inflammation on the alveolar bone tissue. It is worth mentioning that caution is necessary when analyzing our results. Although the results of the present study are promising, they should not be extrapolated to humans, but they open perspectives for future controlled and randomized clinical studies to establish effective and safe therapies for the management of periodontitis during and after treatment with antiresorptive drugs, also aiming to minimize the risk of MRONJ.

## 4. Materials and Methods

### 4.1. Animals, Sample Calculation, Randomization, and Ethics

The present study used forty-eight 6-month-old female rats (Wistar—*Rattus norvegicus*) with a mean body weight of 300 g. The Central Animal Facilities of the São Paulo State University, School of Dentistry (FOA-UNESP, Brazil), supplied the animals. Four rats were housed per cage, receiving food and water ad libitum and maintained under the following conditions: 12 h light/dark cycles, room temperature of 22 ± 2 °C, ventilation and exhaust system allowing for 20 air changes per hour, and relative humidity of 55 ± 5%. Manipulation and experimental procedures were performed according to ARRIVE (Animal Research: Reporting of In Vivo Experiments) and Brazilian National Counseling of Animal Experimentation Control (CONCEA), and the experimental protocol was approved by the Ethics Committee on Animal Use at the School of Dentistry of Araçatuba (593-2020).

The sample size was calculated using Bioestat^®^ software 5.3 (version 5.3; Mamiruá Institute, Tefé, AM, Brazil) to ensure a power greater than 90% (α of 5%; type B error of 20%). It was established that each treatment requires a minimum of 7 repetitions. Therefore, considering any complications throughout the experimental period, we established 8 animals in each group.

This study was conducted using a controlled, randomized, and blind design. Each animal was assigned a number from 1 to 48. Minitab^®^ 17 software (Minitab Inc., State College, PA, USA) was used to create a table that showed the random distribution of the numbered animals among the various experimental groups.

### 4.2. Anesthesia

For all procedures involving pain or discomfort (ovariectomy, ligature installation and removal, local therapy, and euthanasia), animals were anaesthetized using intramuscular injection of ketamine chloride (80 mg/Kg; Syntec, São Paulo, SP, Brazil) and xylazine chloride (6 mg/Kg; Syntec).

### 4.3. Ovariectomy

**Figure 7:**
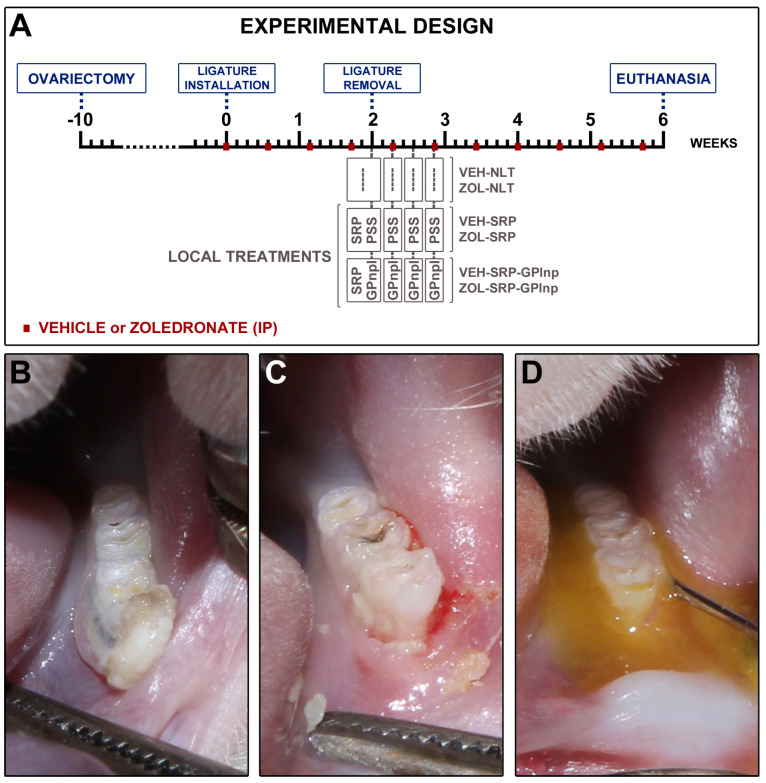
Study design: (**A**) scheme depicting the procedures performed over time in the experimental groups; (**B**) clinical aspect two weeks after ligature placement; (**C**) clinical aspect after the removal of the ligature; (**D**) local use of green propolis-loaded lipid nanoparticles.

All rats underwent bilateral ovariectomy ten weeks before the start of the experiment (Figure 7). After asepsis of the region with 10% povidone-iodine (Rioquímica, São Paulo, SP, Brazil), bilateral incisions were made, and the distal portions of the uterine horns were exposed to remove the ovaries. The uterine tubes were then repositioned and sutured in layers. Vaginal smears were collected throughout the entire ninth week after ovariectomy to confirm its effectiveness. Samples were collected between 7 am and 9 am for 7 consecutive days and analyzed using an optical microscope (AxioLab^®^, Carl Zeiss, Gottingen, Germany). The rats progressed to the following stages of the experiment after confirming persistent diestrus.

### 4.4. Ligature-Induced Periodontitis

On day 0, a cotton ligature (#24; Coats Corrente Ltd., São Paulo, SP, Brazil) was installed around the cervical portion of the left first mandibular molar and securing it with a surgical knot (Figure 7). Ligatures were retained in situ for two weeks to induce EP [42,65,66].

### 4.5. Drug Regimen

Treatment with vehicle (VEH) or zoledronate (ZOL) was performed every four days over six weeks. The VEH administration consisted of intraperitoneal injection of 0.45 mL of 0.9% NaCl solution. Administration of ZOL (Sigma-Aldrich Co., Ltd., St. Loius, MO, USA) consisted of intraperitoneal injection of 100 µg/kg, diluted in 0.45 mL of 0.9% NaCl (Sigma-Aldrich) solution. This dosage and the drug treatment plan consisted of an adaptation for rats of the clinical protocol used to complement oncological therapy in humans [43].

### 4.6. Experimental Groups

On day 14, the ligature was removed, and the animals were randomly distributed in six experimental groups: VEH-NLT (n = 8); ZOL-NLT (n = 8); VEH-SRP (n = 8); ZOL-SRP (n = 8); VEH-SRP-GPlnp (n = 8); and ZOL-SRP-GPlnp (n = 8). The VEH-NLT and ZOL-NLT groups received no local treatment. The VEH-SRP and ZOL-SRP groups received SRP and irrigations with physiological saline. The VEH-SRP-GPlnp and ZOL-SRP-GPlnp groups received SRP and irrigations with GPlnp (Figure 7).

### 4.7. Scaling and Root Planing

After the ligatures were removed, the root surfaces of the ligated molars were manually cleaned using mini-five 1–2 curettes (Hu-Friedy^®^, Chicago, IL, USA). The periodontal debridement was performed by a skilled operator who meticulously executed ten distal-mesial traction movements on the buccal and lingual surfaces and molar furcation [17,18,19].

### 4.8. Green Propolis-Loaded Lipid Nanoparticles

The APISVIDA company (São Paulo, SP, Brazil) gently conceded green propolis extract. Thirty grams of green propolis was added to 70% ethanol (Synth^®^, São Paulo, SP, Brazil), with the final volume adjusted to 100 mL. The mixture was protected from light and subjected to moderate agitation. After one week, the extract was filtered and dried by lyophilization. Green propolis extract was encapsulated in lipid nanoparticles. To obtain a high solid content of this extract, a lipid nanoparticle (microemulsion) composed of linseed oil (Croda^®^, São Paulo, SP, Brazil), green propolis extract (11% *w*/*v*), polyoxyl 15 hydrostearate (BASF^®^, São Paulo, SP, Brazil), and water. The aqueous phase, composed of water with or without surfactant, was poured into the oily phase containing linseed oil and green propolis extract with or without surfactant, under magnetic stirring. The system was kept under agitation for 8 h.

The evaluation of the size, polydispersity index (PDI), and zeta potential (ZP) of the nanoparticles were performed by dynamic light scattering (DLS) using a Zetasizer NanoZS90 (Malvern Panalytical Ltd., Malvern, UK). The measurements were carried out with samples diluted in 1 mM KCl (Synth^®^) solution. To evaluate the encapsulation efficacy of the propolis markers, artepelin C and Baccarin, the dispersion of GPlnp was centrifuged at 5000× *g* in a Microcon ultrafiltration system with a filtration membrane with a molar mass cutoff of 10,000 g/mol (Millipore^®^, Bedford, MA, USA). The concentration of compounds in the filtrate was quantified by ultra-efficiency liquid chromatography coupled to mass spectrometry, with a Single Quadrupole detector (SQ Detector 2, Waters^®^, Milford, MA, USA) with negative electrospray ionization (ESI-). The encapsulation efficacy (EnEf) was calculated by the following equation: EnEf (%) = {[artepelin C or baccarin]_initial_ − [artepelin C or baccarin]_filtered_)/[artepelin C or baccarin]_initial_} × 100.

The morphology of GPlnp was analyzed by transmission electron microscope (TEM) (JEM-100 CXII, JEOL Ltd., Tokyo, Japan). A small amount of the GPlnp dispersion was deposited onto 200-mesh copper grids. A 1% uranyl acetate (Sigma-Aldrich) solution was then applied, followed by allowing the sample to dry at ambient temperature prior to analysis.

### 4.9. Subgingival Irrigation with Physiological Saline Solution or GPlnp

Subgingival irrigations were performed immediately after SRP, and at 2, 4, and 6 days after SRP. The VEH-SRP and ZOL-SRP groups were submitted to irrigation with physiological saline solution irrigation. The VEH-SRP-GPlnp and ZOL-SRP-GPlnp groups received GPlnp irrigation following the same protocol (Figure 7). Subgingival irrigation was carried out using a syringe filled with 1 mL of physiological saline solution or 1 mL of GPlnp. The bevel of the needle was removed to prevent injury to the area before flushing with a solution. The needle tip was directed toward the periodontal pocket. After irrigation, the irrigating solutions remained on the EP site for 5 min. After, the excess was removed with the help of a sterile cotton pallet. To prevent the animals from swallowing the irrigating solutions, they were kept in a horizontal position, and a cotton barrier was placed in the back of the oral cavity.

### 4.10. Euthanasia and Sample Collection

On day 42, euthanasia was performed (Figure 7). The animals were submitted to transcardiac perfusion with 100 mL of 0.9% NaCl solution and 100 μL of heparin, followed by 800 mL of 4% formaldehyde (Sigma-Aldrich) in phosphate-buffered saline (PBS) (Sigma-Aldrich), 0.1M, pH 7.4, 4 °C. After, the left hemimandibles were carefully removed and post fixed with the same solution for 48 h.

### 4.11. Histological Processing

The hemimandibles were demineralized in PBS with 10% ethylene-diaminetetraacetate acid (EDTA) (Sigma-Aldrich) for 60 days. The specimens were dehydrated, diaphanized, and embedded in paraffin. Histological slices, 4μm thick, were obtained from vestibular to lingual. Histological sections of the central region of the furcation were collected in silanized slides. Part of the samples were stained with hematoxylin (Sigma-Aldrich) and eosin (Sigma-Aldrich) (HE) for histological and histometric analysis of the percentage of total bone tissue (PTBT) and percentage of non-vital bone tissue (PNVBT), and part was used for immunohistochemistry.

### 4.12. Immunohistochemistry Processing

The immunohistochemical processing followed the protocol described previously by Souza et al. (2024) [43]. The histological slices were divided into three batches, and each one was incubated for 24 h with one of the following primary antibodies: rabbit alfa tumoral necrosis factor (TNFα) antibody (orb11495, 1:100; Biorbyt, Cambridge, UK); rabbit interleukin 1-beta (IL1-β) antibody (orb382131, 1:100; Biorbyt); and mouse tartrate-resistant acid phosphatase (TRAP) antibody (sc376875, 1:200; Santa Cruz Biotechnology, Dallas, TX, USA). For signal amplification, biotinylated horse anti-mouse/rabbit IgG antibody (1:100; Vector Laboratories, Newark, CA, USA) was used for 2 h, and streptavidin–HRP (1:100; Vector Laboratories) was used for 2 h. The presence of the HRP enzyme was detected using 3,3′-diaminobenzidine tetrahydrochloride (DAB) (ImmPACT DAB Substrate kit, peroxidase, Vector Laboratories) for 1 min. No counterstaining was performed for the slices immunolabeled with TNFα and IL-1β. TRAP slices were counterstained with Harris hematoxylin (Sigma-Aldrich). For negative controls, the primary antibodies were omitted.

## 5. Analysis of the Results

### 5.1. General Health and Intraoral Condition of the Animals

The general health condition of the animals was observed throughout the experimental period and body weight monitored periodically. After euthanasia, a thorough visual inspection of the oral cavity, especially the sites with EP, was performed.

### 5.2. Histopathological Analyses

A certified histologist (EE) who was calibrated and blinded to the groups conducted the histopathological analyses. The following were evaluated: intensity of the local inflammatory response, extension of the inflammatory infiltrate, pattern of structuration of the connective tissue, and alveolar bone in the furcation region. Table 1 presents the parameters and scores based on the histopathological analysis of periodontal tissues.

### 5.3. Histometric Analysis of the Percentage of Total Bone Tissue (PTBT)

Images of the furcation region underwent HE staining and were captured at 200× magnification using a digital camera (AxioCam^®^, Carl Zeiss, Gottingen, Germany) connected to an optical microscope (AxioLab^®^, Carl Zeiss) and a microcomputer with the ZEN2^®^ software (Blue edition; version 6.1.7601; Carl Zeiss). Measurements were performed using ImageJ^®^ software (version 1.5i; National Institute of Health, Bethesda, MD, USA). Three equidistant histological sections were used to measure the percentage of total bone tissue in the furcation region (PTBT). To calculate the PTBT, the total furcation area (TFA) was measured, followed by the area occupied by bone tissue (ABT), both in mm^2^. The apical border of the TFA was defined by drawing a straight line from the mesial root’s apex to the distal root’s apex. Then, the entire outline of the outer surface of the cementum between the roots was traced along this line. To measure the ABT, the same apical limit as the TFA was used, and from this, the entire contour of the external surface of the bone tissue between the roots was followed. PTBT was calculated by multiplying ABT by 100 and dividing by TFA [PTBT = (ABT × 100)/TFA] [67,68]. PTBT is expressed as mean percentage ± standard deviation.

### 5.4. Histometric Analysis of the Percentage of Non-Vital Bone Tissue (PNVBT)

The PNVBT was measured using three equidistant histological sections. Initially, the ABT and the area occupied by non-vital bone tissue (ANVBT) were measured in mm^2^. The PNVBT was then calculated by multiplying ANVBT by 100 and dividing by ABT [PNVBT = (ANVBT × 100)/ABT]. PNVBT is expressed as the mean percentage ± standard deviation. Non-vital bone tissue is defined as areas where contiguous lacunae were present without osteocytes and/or containing only remnants of such cells within the demarcated region(s) of ABT [42,65,66].

### 5.5. Immunolabeling for TNFα, IL-1β, and TRAP in the Furcation Area

Images of histological sections immunolabeling for TNFα, IL-1β, and TRAP in the furcation area of the lower first molar were obtained as previously described. A region of interest (ROI) consists of a square whose sides were 2000 µm and was positioned in the center of the interradicular septum. For TNFα and IL-1β, the coronal limit of this square was the roof of the furcation, which extended apically for a distance of 2000 µm. Using ImageJ^®^ software, the immunolabeling for TNFα and IL-1β was demarcated using the Color Threshold tool to obtain the density of the immunolabeling. The results are expressed in percentage (%) presenting the mean ± standard deviation of each group [43,65,66]. For TRAP, the coronal limit of this square was the alveolar bone crest, which extended apically for a distance of 2000 µm. ImageJ^®^ software was used to quantify TRAP-positive cells. The results were expressed considering the number of cells per mm^2^, showed the mean ± standard deviation for each group [42,43].

### 5.6. Statistical Analysis

Data were analyzed using Bioestat 5.3 (version 5.3; Mamiruá Institute, Tefé, AM, Brazil). PNVBT was considered the primary outcome since bone necrosis as the hallmark MRONJ. Histopathological, PTBT, and immunolabeling for the TNFα, IL-1β, and TRAP analyses were considered secondary outcomes. The normality of data was checked by the Shapiro–Wilk test. For PNVBT, PTBT, TNFα, IL-1β, and TRAP, analysis of variance (ANOVA) was used, followed by the Tukey post-test. For histopathological analyses, nonparametric Kruskal–Wallis analysis and Student–Newman–Keuls post-test were used. Statistical significance was set at *p* < 0.05.

## 6. Conclusions

In conclusion, the local use of GPlnp as an adjuvant therapy to SRP modulated the inflammatory response, reduced substantially the amount of non-vital bone tissue, and improved the tissue repair process. That is, it proved to be both effective and safe in the management of periodontitis in ovariectomized rats treated with an oncological dosage of zoledronate.

## Figures and Tables

**Table 1:** The parameters, scores, and distribution of the specimens based on the histopathological analyses of the furcation region of mandibular left molars in the experimental groups.

HISTOPATHOLOGICAL ANALYSES						
PARAMETERS AND SCORES	QUANTITY OF SPECIMENS					
	EXPERIMENTAL GROUPS					
	VEH-NLT	VEH-SRP	VEH-SRP-GPlnp	ZOL-NLT	ZOL-SRP	ZOL-SRP-GPlnp
**INTENSITY OF LOCAL INFLAMMATORY RESPONSE**						
**(1)** Absence of inflammation	-	-	7	-	-	3
**(2)** Small quantity of inflammatory cells (less than 1/3 of cells were inflammatory cells)	-	7	1	-	-	5
**(3)** Moderate quantity of inflammatory cells (1/3 to 2/3 were inflammatory cells)	5	1	-	3	5	-
**(4)** Large quantity of inflammatory cells (more than 2/3 were inflammatory cells)	3	-	-	5	3	-
**MEDIAN**	**3**	**2 ^†^**	**1 ^†^**	**4 ^‡¶^**	**3 ^‡¶^**	**2 ^†§|^**
**EXTENSION OF INFLAMMATORY INFILTRATE**						
**(1)** Absence of inflammation	-	-	7	-	-	3
**(2)** Partial extension of connective tissue	-	8	1	-	-	5
**(3)** Entire extension of connective tissue, without reaching bone tissue in the furcation region	5	-	-	3	3	-
**(4)** Entire extension of connective tissue and bone tissue	3	-	-	5	5	-
**MEDIAN**	**3**	**2 ^†^**	**1 ^†^**	**4 ^‡¶^**	**4 ^‡¶^**	**2 ^†§|^**
**PATTERN OF STRUCTURATION OF THE CONNECTIVE TISSUE IN THE FURCATION REGION**						
**(1)** Moderate number of fibroblasts and large amount of collagen fibers (dense connective tissue)	-	-	7	-	-	1
**(2)** Moderate amount of both fibroblasts and collagen fibers	-	7	1	-	-	6
**(3)** Small amount of both fibroblasts and collagen fibers	7	1	-	2	2	1
**(4)** Severe tissue disorganization with necrosis areas	1	-	-	6	6	-
**MEDIAN**	**3**	**2**	**1 ^†^**	**4 ^‡¶^**	**4 ^‡¶^**	**2 ^†§|^**
**PATTERN OF STRUCTURATION OF THE ALVEOLAR BONE IN THE FURCATION REGION**						
**(1)** Bone trabeculae with regular contours coated with active osteoblasts, including areas of new bone formation	-	1	8	-	-	-
**(2)** Bone trabeculae with predominantly vital bone tissue, with few areas comprising non-vital bone tissue	8	7	-	-	-	8
**(3)** Bone trabeculae composed of equivalent amounts of vital bone tissue and non-vital bone tissue	-	-	-	3	1	-
**(4)** Bone trabeculae composed predominantly of non-vital bone tissue, with few areas consisting of vital bone tissue	-	-	-	5	7	-
**MEDIAN**	**2**	**2**	**1 ^†‡^**	**4 ^†‡¶^**	**4 ^†‡¶^**	**2 ^¶§|^**

Symbols: †, statistically significant difference compared to the VEH-NLT; ‡, statistically significant difference compared to the VEH-SRP; ¶, a statistically significant difference compared to the VEH-SRP-GPlnp; §, a statistically significant difference compared to the ZOL-NLT; |, a statistically significant difference compared to the ZOL-SRP.

## Data Availability

The data are contained within the article.

## References

[1] Ruggiero S.L., Dodson T.B., Aghaloo T., Carlson E.R., Ward B.B., Kademani D. (2022). American Association of Oral and Maxillofacial Surgeons’ Position Paper on Medication-Related Osteonecrosis of the Jaws-2022 Update. J. Oral. Maxillofac. Surg..

[2] Tetradis S., Allen M.R., Ruggiero S.L. (2023). Pathophysiology of Medication-Related Osteonecrosis of the Jaw-A Minireview. JBMR Plus.

[3] Murphy J., Mannion C.J. (2020). Medication-related osteonecrosis of the jaws and quality of life: Review and structured analysis. Br. J. Oral. Maxillofac. Surg..

[4] Halpern L.R., Adams D.R. (2024). Treatment of medication-related osteonecrosis of the jaw: Controversies in causality and therapy. Dent. Clin. N. Am..

[5] Beth-Tasdogan N.H., Mayer B., Hussein H., Zolk O., Peter J.U. (2022). Interventions for managing medication-related osteonecrosis of the jaw. Cochrane Database Syst. Rev..

[6] AlRowis R., Aldawood A., AlOtaibi M., Alnasser E., AlSaif I., Aljaber A., Natto Z. (2022). Medication-Related Osteonecrosis of the Jaw (MRONJ): A Review of Pathophysiology, Risk Factors, Preventive Measures and Treatment Strategies. Saudi Dent. J..

[7] Kim H.Y. (2021). Review and Update of the Risk Factors and Prevention of Antiresorptive-Related Osteonecrosis of the Jaw. Endocrinol. Metab..

[8] McGowan K., McGowan T., Ivanovski S. (2018). Risk Factors for Medication-Related Osteonecrosis of the Jaws: A Systematic Review. Oral. Dis..

[9] Lorenzo-Pouso A.I., Pérez-Sayáns M., Chamorro-Petronacci C., Gándara-Vila P., López-Jornet P., Carballo J., García-García A. (2020). Association between periodontitis and medication-related osteonecrosis of the jaw: A systematic review and meta-analysis. J. Oral. Pathol. Med..

[10] Huang Y.F., Lin K.C., Liu S.P., Chang C.T., Muo C.H., Chang P.J., Tsai C.H., Wu C.Z. (2022). The association between the severity of periodontitis and osteonecrosis of the jaw in patients with different cancer locations: A nationwide population-based study. Clin. Oral. Investig..

[11] Thumbigere-Math V., Michalowicz B.S., Hodges J.S., Tsai M.L., Swenson K.K., Rockwell L., Gopalakrishnan R. (2014). Periodontal disease as a risk factor for bisphosphonate-related osteonecrosis of the jaw. J. Periodontol..

[12] Williams D.W., Ho K., Lenon A., Kim S., Kim T., Gwack Y., Kim R.H. (2022). Long-Term Ligature-Induced Periodontitis Exacerbates Development of Bisphosphonate-Related Osteonecrosis of the Jaw in Mice. J. Bone Miner. Res..

[13] Messer J.G., Mendieta Calle J.L., Jiron J.M., Castillo E.J., Van Poznak C., Bhattacharyya N., Kimmel D.B., Aguirre J.I. (2018). Zoledronic Acid Increases the Prevalence of Medication-Related Osteonecrosis of the Jaw in a Dose Dependent Manner in Rice Rats (*Oryzomys palustris*) with Localized Periodontitis. Bone.

[14] Aghaloo T.L., Kang B., Sung E.C., Shoff M., Ronconi M., Gotcher J.E., Bezouglaia O., Dry S.M., Tetradis S. (2011). Periodontal disease and bisphosphonates induce osteonecrosis of the jaws in the rat. J. Bone Miner. Res..

[15] Cobb C.M., Sottosanti J.S. (2021). A re-evaluation of scaling and root planing. J. Periodontol..

[16] Suvan J., Leira Y., Moreno Sancho F.M., Graziani F., Derks J., Tomasi C. (2020). Subgingival instrumentation for treatment of periodontitis. A systematic review. J. Clin. Periodontol..

[17] Mello-Neto J.M., Ervolino E., Elangovan G., Toro L.F., Lee J., Gustafsson A., Figueredo C.M.D.S. (2023). The Resolution of Periodontal Inflammation Promotes Changes in Cytokine Expression in the intestine and Gingival Tissues of Aged Rats with DSS-Induced Colitis. J. Clin. Med..

[18] Silveira G.R.C., de Lima D.C., Cintra L.T.A., Brigagão M.R.P.L., Ervolino E., Fernandes L.A. (2022). Systemic and local effects of doxycycline and low-intensity laser treatment on periodontitis in rats. J. Periodontal Implant. Sci..

[19] Silveira G.R.C., de Lima D.C., Cintra L.T.A., Brigagão M.R.P.L., Ervolino E., Fernandes L.A. (2021). Influence of Doxycycline and InGaAlP Diode Laser at 660 nm Wavelength in the Treatment of Periodontitis Induced in Rats: In Vivo Study. Photochem. Photobiol..

[20] Diniz-Freitas M., Fernández-Feijoo J., Diz Dios P., Pousa X., Limeres J. (2018). Denosumab-related osteonecrosis of the jaw following non-surgical periodontal therapy: A case report. J. Clin. Periodontol..

[21] Braun E., Iacono V.J. (2006). Bisphosphonates: Case report of nonsurgical periodontal therapy and osteochemonecrosis. Int. J. Periodontics Restor. Dent..

[22] Araujo N.J. (2017). Experimental Periodontitis in Rats Treated with an Oncological Dose of Zoledronate: Analysis of Disease Progression and Evaluation of Periodontal Response to Conventional Mechanical Treatment. Master’s Thesis.

[23] Eghbali Zarch R., Askari M., Boostani H., Mirzaii-Dizgah I. (2021). Effect of propolis extract on clinical parameters and salivary level of matrix metalloproteinase 8 in periodontitis patients: A randomized controlled clinical trial. J. Adv. Periodontol. Implant. Dent..

[24] Nakao R., Senpuku H., Ohnishi M., Takai H., Ogata Y. (2020). Effect of Topical Administration of Propolis in Chronic Periodontitis. Odontology.

[25] Toker H., Ozan F., Ozer H., Ozdemir H., Eren K., Yeler H. (2008). A morphometric and histopathologic evaluation of the effects of propolis on alveolar bone loss in experimental periodontitis in rats. J. Periodontol..

[26] Anjum S.I., Ullah A., Khan K.A., Attaullah M., Khan H., Ali H., Bashir M.A., Tahir M., Ansari M.J., Ghramh H.A. (2019). Composition and functional properties of propolis (bee glue): A review. Saudi J. Biol. Sci..

[27] dos Santos F.F., Morais-Urano R.P., Cunha W.R., de Almeida S.G., Cavallari P.S.D.S.R., Manuquian H.A., Pereira H.A., Furtado R., Santos M.F.C., Amdrade e Silva M.L. (2022). A review on the anti-inflammatory activities of Brazilian green, brown and red propolis. J. Food Biochem..

[28] Weis W.A., Ripari N., Conte F.L., da Silva Honorio M., Sartori A.A., Matucci R.H., Sforcin J.M. (2022). An overview about apitherapy and its clinical applications. Phytomedicine Plus.

[29] Sforcin J.M. (2016). Biological Properties and Therapeutic Applications of Propolis. Phytother. Res..

[30] Liu Y., Liang Y., Yuhong J., Xin P., Han J.L., Du Y., Yu X., Zhu R., Zhang M., Chen W. (2024). Advances in Nanotechnology for Enhancing the Solubility and Bioavailability of Poorly Soluble Drugs. Drug Des. Dev. Ther..

[31] Soni A.K., Jha R.K. (2024). Nanotechnology’s Applications and Potential in Various Fields. Cureus.

[32] Mercan D.A., Niculescu A.G., Grumezescu A.M. (2022). Nanoparticles for Antimicrobial Agents Delivery—An Up-to-Date Review. Int. J. Mol. Sci..

[33] Li J., Wang Y., Tang M., Zhang C., Fei Y., Li M., Li M., Gui S., Guo J. (2024). New insights into nanotherapeutics for periodontitis: A triple concerto of antimicrobial activity, immunomodulation and periodontium regeneration. J. Nanobiotechnol..

[34] Li L., Qu J., Liu W., Peng B., Cong S., Yu H., Zhang B., Li Y. (2024). Advancements in Characterization Techniques for Microemulsions: From Molecular Insights to Macroscopic Phenomena. Molecules.

[35] Tartaro G., Mateos H., Schirone D., Angelico R., Palazzo G. (2020). Microemulsion Microstructure(s): A Tutorial Review. Nanomaterials.

[36] Khuda F., Baharin B., Anuar N.N.M., Satimin B.S.F., Nasruddin N.S. (2024). Effective Modalities of Periodontitis Induction in Rat Model. J. Vet. Dent..

[37] Staubli N., Schmidt J.C., Rinne C.A., Signer-Buset S.L., Rodriguez F.R., Walter C. (2019). Animal Experiments in Periodontal and Peri-Implant Research: Are There Any Changes?. Dent. J..

[38] Graves D.T., Kang J., Andriankaja O., Wada K., Rossa C. (2012). Animal models to study host-bacteria interactions involved in periodontitis. Front. Oral. Biol..

[39] Hadad H., Matheus H.R., Pai S.I., Souza F.A., Guastaldi F.P.S. (2024). Rodents as an animal model for studying tooth extraction-related medication-related osteonecrosis of the jaw: Assessment of outcomes. Arch. Oral. Biol..

[40] Aguirre J.I., Castillo E.J., Kimmel D.B. (2021). Biologic and pathologic aspects of osteocytes in the setting of medication-related osteonecrosis of the jaw (MRONJ). Bone.

[41] Kuroshima S., Go V.A., Yamashita J. (2012). Increased numbers of nonattached osteoclasts after long-term zoledronic acid therapy in mice. Endocrinology.

[42] Ervolino E., Olivo M.B., Toro L.F., Freire J.O.A., Ganzaroli V.F., Guiati I.Z., Nuernberg M.A.A., Franciscon J.P.S., Cintra L.T.A., Garcia V.G. (2022). Effectiveness of antimicrobial photodynamic therapy mediated by butyl toluidine blue in preventing medication-related osteonecrosis of the jaws in rats. Photodiagnosis Photodyn. Ther..

[43] Souza E.Q.M., Toro L.F., Ganzaroli V.F., de Oliveira Alvarenga Freire J., Matsumoto M.A., Casatti C.A., Cintra L.T.A., Buchaim R.L., Issa J.P.M., Garcia V.G. (2024). Peri-implantitis increases the risk of medication-related osteonecrosis of the jaws associated with osseointegrated implants in rats treated with zoledronate. Sci. Rep..

[44] Almeida-Junior S., de Oliveira K.R.P., Marques L.P., Martins J.G., Ubeda H., Santos M.F.C., Rodrigues M.A., Andrade e Silva M.L., Ambrósio S.R., Bastos J.K. (2024). In vivo anti-inflammatory activity of BACCHARIN from BRAZILIAN green PROPOLIS. Fitoterapia.

[45] Beserra F.P., Gushiken L.F.S., Hussni M.F., Ribeiro V.P., Bonamin F., Jackson C.J., Pellizzon C.H., Bastos J.K. (2021). Artepillin C as an outstanding phenolic compound of Brazilian green propolis for disease treatment: A review on pharmacological aspects. Phytother. Res..

[46] Ferreira J.C., Reis M.B., Coelho G.D.P., Gastaldello G.H., Peti A.P.F., Rodrigues D.M., Bastos J.K., Campo V.L., Sorgi C.A., Faccioli L.H. (2021). Baccharin and p-coumaric acid from green propolis mitigate inflammation by modulating the production of cytokines and eicosanoids. J. Ethnopharmacol..

[47] Gomes K.O., Messias da Silva L.C.F., dos Santos R.D., Prado B.A., da Silva Montes P., Silva Rodrigues L.F., de Araújo M.O., Bilac C.A., Freire D.O., Gris E.F. (2024). Chemical characterization and antibacterial activities of Brazilian propolis extracts from Apis mellifera bees and stingless bees (*Meliponini*). PLoS ONE.

[48] Jenny J.C., Kuś P.M., Szweda P. (2024). Investigation of antifungal and antibacterial potential of green extracts of propolis. Sci. Rep..

[49] Veiga R.S., de Mendonça S., Mendes P.B., Paulino N., Mimica M.J., Lagareiro Netto A.A., Lira I.S., López B.G., Negrão V., Marcucci M.C. (2017). Artepillin C and phenolic compounds responsible for antimicrobial and antioxidant activity of green propolis and Baccharis dracunculifolia DC. J. Appl. Microbiol..

[50] de Sá Assis M.A., de Paula Ramos L., Abu Hasna A., de Queiroz T.S., Pereira T.C., Nagai de Lima P.M., Berretta A.A., Marcucci M.C., Talge Carvalho C.A., de Oliveira L.D. (2022). Antimicrobial and Antibiofilm Effect of Brazilian Green Propolis Aqueous Extract against Dental Anaerobic Bacteria. Molecules.

[51] Figueiredo L.C., Freitas Figueiredo N., da Cruz D.F., Baccelli G.T., Sarachini G.E., Bueno M.R., Feres M., Bueno-Silva B. (2022). Propolis, Aloe Vera, Green Tea, Cranberry, Calendula, Myrrha and Salvia Properties against Periodontal Microorganisms. Microorganisms.

[52] Vadillo-Rodríguez V., Cavagnola M.A., Pérez-Giraldo C., Fernández-Calderón M.C. (2021). A physico-chemical study of the interaction of ethanolic extracts of propolis with bacterial cells. Colloids Surf. B Biointerfaces.

[53] Przybyłek I., Karpiński T.M. (2019). Antibacterial Properties of Propolis. Molecules.

[54] Bryan J., Redden P., Traba C. (2016). The mechanism of action of Russian propolis ethanol extracts against two antibiotic-resistant biofilm-forming bacteria. Lett. Appl. Microbiol..

[55] Islam S., Hussain E.A., Shujaat S., Khan U.M., Ali Q., Malook S.U., Ali D. (2024). Antibacterial potential of Propolis: Molecular docking, simulation and toxicity analysis. AMB Express..

[56] Wood P.L. (2024). Metabolic and Lipid Biomarkers for Pathogenic Algae, Fungi, Cyanobacteria, Mycobacteria, Gram-Positive Bacteria, and Gram-Negative Bacteria. Metabolites.

[57] Yoshimasu Y., Ikeda T., Sakai N., Yagi A., Hirayama S., Morinaga Y., Furukawa S., Nakao R. (2018). Rapid Bactericidal Action of Propolis against Porphyromonas gingivalis. J. Dent. Res..

[58] Sun H., Li P., Kong Q., Deng F., Yu X. (2023). Zoledronic acid affects the process of Porphyromonas gingivalis infecting oral mucosal epithelial barrier: An in-vivo and in-vitro study. Front. Cell Infect. Microbiol..

[59] Shang J., Liu H., Zheng Y., Zhang Z. (2023). Role of oxidative stress in the relationship between periodontitis and systemic diseases. Front. Physiol..

[60] Bagan J., Sáez G.T., Tormos M.C., Gavalda-Esteve C., Bagan L., Leopoldo-Rodado M., Calvo J., Camps C. (2014). Oxidative stress in bisphosphonate-related osteonecrosis of the jaws. J. Oral. Pathol. Med..

[61] Yang J., Pi A., Yan L., Li J., Nan S., Zhang J., Hao Y. (2022). Research Progress on Therapeutic Effect and Mechanism of Propolis on Wound Healing. Evid. Based Complement. Alternat. Med..

[62] Salrian A.A., Behzadi A., Oloumi M.M., Farajli Abbasi M., Delshad S., Moghadaszadeh M. (2022). Amplification of Wound Healing by Propolis and Honey Ointment in Healthy and Diabetic Rat Models; Histopathological and Morphometric Findings. Arch. Razi Inst..

[63] Furukawa M., Wang J., Kurosawa M., Ogiso N., Shikama Y., Kanekura T., Matsushita K. (2021). Effect of green propolis extracts on experimental aged gingival irritation in vivo and in vitro. J. Oral. Biosci..

[64] Cho Y.D., Kim K.H., Lee Y.M., Ku Y., Seol Y.J. (2021). Periodontal wound healing and tissue regeneration: A narrative review. Pharmaceuticals.

[65] Toro L.F., de Mello-Neto J.M., Santos F.F.V.D., Ferreira L.C., Statkievicz C., Cintra L.T.A., Issa J.P.M., Dornelles R.C.M., de Almeida J.M., Nagata M.J.H. (2019). Application of Autologous Platelet-Rich Plasma on Tooth Extraction Site Prevents Occurrence of Medication-Related Osteonecrosis of the Jaws in Rats. Sci. Rep..

[66] Ervolino E., Statkievicz C., Toro L.F., de Mello-Neto J.M., Cavazana T.P., Issa J.P.M., Dornelles R.C.M., de Almeida J.M., Nagata M.J.H., Okamoto R. (2019). Antimicrobial photodynamic therapy improves the alveolar repair process and prevents the occurrence of osteonecrosis of the jaws after tooth extraction in senile rats treated with zoledronate. Bone.

[67] Novaes V.C.N., Ervolino E., Fernandes G.L., Cunha C.P., Theodoro L.H., Garcia V.G., de Almeida J.M. (2022). Influence of the Treatment with the Antineoplastic Agents 5-Fluorouracil and Cisplatin on the Severity of Experimental Periodontitis in Rats. Support. Care Cancer.

[68] Garcia V.G., Novaes V.C., de Almeida J.M., Longo M., Ervolino E., Bomfim S.R., Theodoro L.H. (2015). Evaluation of the progression and treatment of experimental periodontitis in rats subjected to chemotherapy with 5-fluorouracil. Support. Care Cancer.

